# Ultra-Processed Food Consumption Among Chilean Preschoolers Is Associated With Diets Promoting Non-communicable Diseases

**DOI:** 10.3389/fnut.2021.601526

**Published:** 2021-03-26

**Authors:** C. Araya, C. Corvalán, G. Cediel, L. S. Taillie, M. Reyes

**Affiliations:** ^1^Institute of Nutrition and Food Technology (INTA), University of Chile, Santiago, Chile; ^2^Escuela de Nutrición y Dietética, Facultad de Salud, Universidad Santo Tomás, Santiago, Chile; ^3^Department of Nutrition, University of São Paulo, São Paulo, Brazil; ^4^School of Nutrition and Dietetics, University of Antioquia, Medellín, Colombia; ^5^Department of Nutrition, Carolina Population Center, Gillings School of Global Public Health, University of North Carolina, Chapel Hill, NC, United States

**Keywords:** ultra-processed foods, children, preschooler, diet, Chile

## Abstract

**Introduction:** In adults, intake of ultra-processed foods (UPF) has been linked with poor diets and adverse health outcomes. In young children, evidence is scarcer but suggests a higher dietary share of UPF.

**Objective:** To quantify the intake of UPF and its association with the nutrient composition of the diet in a sample of preschoolers in Santiago, Chile.

**Methods:** Cross-sectional analysis of dietary data (24-h recall survey) from 960 preschoolers. Foods were categorized according to the extent and purpose of processing (NOVA classification) and participants were classified in quintiles of UPF intake. We explored the associations between UPF intake (% of the total energy) and intake of nutrients of concern for non-communicable disease development (carbohydrates, total sugars, fats, and sodium), and nutrients for promotion (proteins, polyunsaturated fats, iron, calcium, zinc, vitamins A, D, C, and B_12_, folate, and fiber) using multivariate regression after controlling for covariates.

**Results:** UPF constituted 49% of the total energy intake. Preschoolers with higher intake consumed more energy, saturated and monounsaturated fats, carbohydrates, total sugars, and vitamin D, compared to preschoolers in the lowest quintile of UPF intake. In contrast, UPF intake was negatively associated with the consumption of proteins, polyunsaturated fats, fiber, zinc, vitamin A, and sodium (*p* < 0.05).

**Conclusion:** In Chilean preschoolers, UPF was the primary source of energy intake. The dietary share of UPF was associated with the nutrient composition of the diet. Improving children's diet should consider not only promoting healthy food consumption but also limiting UPF consumption.

## Introduction

Unhealthy diets—characterized by a low intake of whole grains, fruits, nuts and seeds, seafood, and legumes together with a high intake of sodium, trans fats, processed meats, and sugar-sweetened beverages, among other factors—account for 22% of deaths and 15% of disability-adjusted life years (DALYs) (1, 2).

Over the last few decades, in several countries, traditional foods have been replaced by ultra-processed foods (UPF); products manufactured industrially using processes and ingredients not commonly used in culinary preparations (3, 4). UPF consumption has been associated with a variety of markers of poor nutritional quality. A greater dietary share of UPF has been linked to diets promoting non-communicable diseases (NCD), characterized by a high content of energy and nutrients of concern such as sugars, saturated fats, trans fats, and sodium, as well as poor content of micronutrients and fiber among different populations (5–9). Different longitudinal studies among adults have shown an association between UPF consumption and the development of NCDs such as overweight/obesity, high blood pressure, and cancer (10–12). These conditions, in turn, are associated with greater risks of mortality (13).

Conversely, evidence of the link between UPF consumption and the nutrient composition of the diet or health impact among children is scarce (14–18). Studies on UPF consumption in children indicate that the intake of these products is higher than that in the general population (19, 20). Chile, a post-nutrition transitional country where approximately 75% of the adults and 50% of children aged 6–7 years are either overweight or obese (21–23), is no exception. In 2010, the National Dietary Survey showed a UPF consumption of 29% among the general population and 38% among children and adolescents (i.e., 2–19 years old) (20). In June 2016, the Chilean government implemented a set of regulations aimed at improving diets and preventing obesity, particularly in children (24, 25). Although the regulations were not directed to decrease UPF intake, the foods targeted were those with added sugars, fats, or sodium as part of their processing (25) and, therefore, encompassed most UPF. Understanding UPF intake among young children and its association with the intake of nutrients of concern for NCD development and nutrients for promotion prior to the implementation of the regulation is important for evaluating whether this set of policies may reduce UPF intake and lead to improvements in health. To address these objectives, we analyzed detailed dietary information collected from preschool children of middle to low socioeconomic status who participated in a longitudinal study in Santiago, Chile.

## Materials and Methods

### Participants and Setting

During the first semester of 2016, the mothers of preschoolers attending public schools of the Chilean National School Board Program (JUNAEB) were invited to participate in the study. These counties are primarily of middle to low socioeconomic status (26). The inclusion criteria for the children were as follows: an age of 4–6 years, born from a single pregnancy, without any gastrointestinal condition that could affect food intake or growth, and with a mother responsible for food purchases and childcare in the home. The participants were recruited via written and verbal announcements to the guardians of students enrolled in kindergarten and preschool. We evaluated 962 mother-child pairs who met the inclusion criteria, two of which were excluded due to lack of plausible anthropometric data or incomplete dietary survey data. Thus, the analyses included 960 mother-child pairs, with approximately 190 participants per group when the sample was divided into quintiles according to the distribution of UPF intake. This sample size allowed the detection of differences between the 1st and 5th quintiles of a 1/3 of the standard deviation (SD) of the outcome, which is in line with the differences detected in a secondary analysis of the National Dietary Survey from 2010, were a quintile of 20% of children and adolescents (aged 2–19 years) with the lowest UPF intake had an average added sugars intake of 8 ± 6% of the energy intake, compared to 16 ± 13% among children and adolescents in the highest quintile of UPF consumption (20). This study was approved by the Ethics Committee of the Institute of Nutrition and Food Technology (INTA) of the Universidad de Chile and by the Institutional Review Board of the University of North Carolina at Chapel Hill. All procedures complied with the principles of the Declaration of Helsinki. Informed consent was obtained from the mothers of all participants.

### Study Description

Between April and August 2016, the participants were evaluated by trained dietitians at either INTA's outpatient clinic or the participants' homes. Anthropometric assessments were performed for preschoolers. Weight and height were collected following standardized procedures and standardized dietitians (intraclass correlation coefficient [ICC] > 0.75 for all measurements). The participants were measured while barefoot and wearing light clothing. Weight was collected using portable electronic scales (Seca 770 or 803, precision of 0.1 kg) and height with portable stadiometers (Seca 217, to the nearest 0.1 cm). The mothers were asked for the duration of their formal education.

### Evaluation of Food and Nutrient Intakes

The mothers of the preschoolers completed a 24-h recall (24 h) survey to estimate all of the foods and beverages consumed by the child the previous day (weekday or weekend), including food consumed at the school or nursery. This survey was conducted according to the United States Department of Agriculture (USDA) Automated Multiple-Pass Method (27). To more accurately estimate the proportions of consumed foods, the photographic atlas of food proportions used in the National Dietary Survey was employed (28). The mothers were specifically asked about the use of table salt. All data were recorded and analyzed using the 24 h survey software which considered 4,644 foods and 873 recipes commonly consumed by the Chilean population (including those provided by the school feeding program), with standardized ingredients and quantities (29–31).

### Energy and Nutritional Contents of the Foods

Given that a local database for the chemical and nutritional compositions of foods and beverages is not available for Chile, the energy and nutrient contents were assigned based on the USDA Food Composition Database, Release 28 (32). Foods from the USDA database were selected based on information provided by the *Guide on the Nutritional Composition of Natural Foods and Common Chilean Preparations* (33) as well as data declared as part of the nutritional information on packaged products (collected during 2015–2016 for packaged products available at supermarkets) (34). Most similar food (20% maximum variation) was selected based on the content of energy, macronutrient (i.e., proteins, carbohydrates, total fats), saturated fats, total sugars, and sodium. Information on other nutrients (i.e., other subtypes of fats, fiber, vitamins, and minerals) was not considered when selecting the most similar food from the USDA database.

### Classification of Foods by the Degree of Processing

The 4,644 reported foods, including preparations (e.g., natural milk with sugar, cooked rice, and lentil stew, among others), were categorized into one of four groups and then into subgroups according to the NOVA classification system (35, 36) as follows:

- Group 1, Unprocessed or minimally processed food: natural foods altered by processes such as drying, freezing, pasteurization, among others that do not add substances such as salt, sugar, oil, or fats to the original food; comprising 13 subgroups (e.g., milk and plain yogurt; fruits; legumes; meat).
- Group 2, Processed culinary ingredients: substances obtained directly from group 1 foods or from nature by processes such as pressing, refining, and milling, among others; comprising five subgroups (e.g., plant oils, table sugar, table salt).
- Group 3, Processed foods: relatively simple products made by adding sugars, oil, salt, or other group 2 substances to group 1 foods, with the main purpose of increasing the durability of group 1 foods or to modify their sensory qualities; comprising five subgroups (e.g., breads [fresh, unpackaged]; cheese; vegetables, fruits, and other plant foods preserved in brine or syrup).
- Group 4, Ultra-processed foods: industrial formulations typically with five or more ingredients, including the formulations used in group 3 and additives as anti-oxidants, stabilizers, preservatives, as well as substances not commonly used in culinary preparations whose purpose is to imitate the sensory qualities of group 1 foods or of culinary preparations of these foods or to disguise the undesirable sensory qualities of the final products; comprising 21 subgroups (e.g., milk-based drinks; sandwiches and hamburgers on a bun [ready-to-eat/heat]; cakes, cookies, and pies).

Detailed descriptions of each subgroup are provided as Supplementary Material.

### Nutritional Outcomes

The nutrient composition of the diet was assessed by the daily intake of energy, total carbohydrates, total sugars, fiber, total fats, fat subtypes (i.e., saturated, monounsaturated, polyunsaturated, and trans), proteins, iron, calcium, zinc, vitamins A, D, C, and B_12_, and folate. The contribution of every NOVA food group was also estimated. The intake was reported (mean and SD) in absolute terms [i.e., grams, milligrams, micrograms, or international units (IU)], considering energy intake (per 100 or 1,000 kcal) and relative to the total daily intake of the specific participant.

### Covariates

Preschoolers' sex (male vs. female) and age were considered as covariates. Body mass index (BMI, weight [kg]/height [m^2^]) and age- and sex-specific z scores were calculated based on the World Health Organization's (WHO) 2006 growth standards for preschoolers <5 years and 2007 references for older preschoolers. The weight status categories were defined according to BMI z score as normal weight (−1 to <1), overweight (+1 to <2), and obese (≥2). Formal education of the mother was categorized as low (<8 years of schooling), medium (8–12 years), and high (>12 years). Finally, the day of the 24 h survey was also included (weekday vs. weekend) as a covariate.

### Statistical Analysis

The participants were grouped into quintiles based on the amount of energy consumed from UPF. The general and anthropometric characteristics of children across quintiles of UPF intake were compared using chi-squared tests and one-way analysis of variance (ANOVA), with Bonferroni *post-hoc* correction. The associations between UPF consumption and the intake of energy and different nutrients were studied using linear regression models, considering quintiles of dietary share of UPF (set of indicator variables) as the predictor, and covariates relevant to the association (i.e., variation of the predictor's beta coefficient >10% when removed). Adjusted means and standard errors derived from the models are presented. The final models met all expected assumptions. *P*-values <0.05 were considered statistically different. All data were analyzed using Stata v13 (StataCorp, TX, USA).

## Results

The sociodemographic characteristics of the sample are summarized in Table 1. Participants were on average 5 years of age and nearly half of the participants were overweight or obese, with an average BMI z score of 1. Most mothers had completed high school (52%) and <10% had a low level of education.

**Table 1:** Sociodemographic variables, weight status, and energy intake among the participants (*n* = 960).

**Variables**	**Descriptive**
Female, *n* (%)	498 (51.9)
Age [years], mean ± SD	4.8 ± 0.5
BMI [z score], mean ± SD^*^	1.0 ± 1.2
**Weight status**, ***n*** (%)^*^	
Normal weight (< −1 to 0.9 SD)	508 (52.9)
Overweight (1 to 1.9 SD)	276 (28.8)
Obese (≥2 SD)	175 (18.3)
**Mother's education level**, ***n*** **(%)**	
Low (<8 years of schooling)	69 (7.3)
Medium (8–12 years of schooling)	499 (52.0)
High (>12 years of schooling)	392 (40.7)

*Values represent either the total number and (percentage) or mean ± standard deviation*.

* *n = 959 with anthropometric data*.

Table 2 displays the intake of energy, macronutrients, micronutrients, and fiber by NOVA food groups. UPF accounted for 49% of the daily energy intake, whereas 32% of energy came from unprocessed or minimally processed foods, 10% from processed foods, and 8% from culinary ingredients. UPF was also the main dietary source of carbohydrates (53%), total sugars (76%), total fats (48%), saturated fats (55%), trans fats (49%), and sodium (39%). In contrast, the groups of minimally processed foods and culinary ingredients were the main sources of proteins (47% from group 1), fiber (51% from group 1), and polyunsaturated fats (43% from group 2).

**Table 2:** Intake of energy and nutrients of concern by the participants (*n* = 960).

	**Total**	**Unprocessed or minimally processed foods**	**Processed culinary ingredients**	**Processed foods**	**Ultra-processed foods**	*p*-value^*^
**Energy intake**						
Absolute [kcal]	1,240.0 ± 392.4	398.9 ± 208.0^a^	98.8 ± 80.3^b^	126.5 ± 125.0^c^	617.5 ± 318.3^d^	<0.01
Relative [% daily energy intake]	100	32.4 ± 14.9^a^	7.9 ± 5.9^b^	10.4 ± 9.9^c^	49.2 ± 18.0^d^	<0.01
**Protein intake**						
Absolute [g]	43.1 ± 15.4	20.9 ± 12.9^a^	0.0 ± 0.1^b^	5.6 ± 6.3^c^	16.6 ± 9.7^d^	<0.01
Absolute [% daily energy intake]	14.1 ± 3.3	6.9 ± 4.0^a^	0.0 ± 0.0^b^	1.8 ± 2.0^c^	5.4 ± 2.7^d^	<0.01
Relative [% daily protein intake]	100	47.3 ± 20.7^a^	0.0 ± 0.1^b^	12.9 ± 13.1^c^	39.8 ± 19.8^d^	<0.01
**Carbohydrate intake**						
Absolute [g]	178.2 ± 58.4	60.2 ± 33.9^a^	3.3 ± 7.7^b^	19.1 ± 19.4^c^	95.7 ± 49.4^d^	<0.01
Absolute [% daily energy intake]	57.8 ± 7.2	19.7 ± 10.0^a^	1.1 ± 2.5^b^	6.4 ± 6.4^c^	30.6 ± 11.8^d^	<0.01
Relative [% daily carbohydrates intake]	100	34.2 ± 17.1^a^	1.8 ± 4.2^b^	11.1 ± 11.0^c^	52.8 ± 18.7^d^	<0.01
**Total sugars intake**						
Absolute [g]	88.2 ± 35.9	15.0 ± 14.0^a^	3.2 ± 7.6^b^	1.6 ± 3.2^b^	68.3 ± 35.5^c^	<0.01
Absolute [% daily energy intake]	28.7 ± 8.4	5.0 ± 4.7^a^	1.1 ± 2.5^b^	0.5 ± 1.0^b^	22.1 ± 9.3^c^	<0.01
Relative [% daily total sugars intake]	100	18.6 ± 17.6^a^	3.7 ± 8.4^b^	2.2 ± 4.5^b^	75.5 ± 19.9^c^	<0.01
**Fiber intake**						
Absolute [g]	11.5 ± 6.6	6.4 ± 5.7^a^	0.0 ± 0.0^b^	1.7 ± 1.4^c^	3.8 ± 2.9^d^	<0.01
Absolute [g/1,000 kcal intake]	9.2 ± 4.3	5.2 ± 4.3^a^	0.0 ± 0.0^b^	1.4 ± 1.1^c^	3.0 ± 2.0^d^	<0.01
Relative [% daily total fiber intake]	100	51.4 ± 23.2^a^	0.0 ± 0.1^b^	17.4 ± 14.6^c^	36.0 ± 21.8^d^	<0.01
**Total fats intake**						
Absolute [g]	40.7 ± 17.3	8.4 ± 7.4^a^	9.6 ± 8.1^b^	3.0 ± 4.5^c^	19.7 ± 13.6^d^	<0.01
Absolute [% daily energy intake]	29.1 ± 6.5	6.2 ± 5.1^a^	6.9 ± 5.4^b^	2.2 ± 2.9^c^	13.9 ± 7.5^d^	<0.01
Relative [% daily total fats intake]	100	21.3 ± 16.6^a^	23.4 ± 16.8^b^	7.6 ± 10.2^c^	47.7 ± 22.7^d^	<0.01
**Saturated fats intake**						
Absolute [g]	13.9 ± 6.8	3.2 ± 3.6^a^	1.7 ± 1.8^b^	1.2 ± 2.1^c^	7.8 ± 5.7^d^	<0.01
Absolute [% daily energy intake]	9.9 ± 3.3	2.3 ± 2.5^a^	1.3 ± 1.2^b^	0.8 ± 1.4^c^	5.5 ± 3.2^d^	<0.01
Relative [% daily saturated fats intake]	100	22.9 ± 20.4^a^	13.7 ± 13.2^b^	8.2 ± 12.9^c^	55.1 ± 25.1^d^	<0.01
**Trans fats intake**						
Absolute [g]	0.3 ± 0.4	0.1 ± 0.2 ^a^	0.0 ± 0.1 ^b^	0.0 ± 0.0 ^c^	0.2 ± 0.3 ^d^	<0.01
Absolute [% daily energy intake]	0.2 ± 0.3	0.1 ± 0.1 ^a^	0.0 ± 0.1 ^b^	0.0 ± 0.0 ^c^	0.1 ± 0.2 ^d^	<0.01
Relative [% daily trans fats intake]	100	34.5 ± 36.5^a^	12.3 ± 24.9^b^	10.9 ± 19.3^c^	49.3 ± 37.7^d^	<0.01
**Monounsaturated fats intake**						
Absolute [g]	13.1 ± 6.3	3.0 ± 2.9^a^	2.8 ± 2.5^a^	1.1 ± 1.7^b^	6.6 ± 5.2^c^	<0.01
Absolute [% daily energy intake]	9.3 ± 2.8	2.2 ± 1.9^a^	2.0 ± 1.7^a^	0.8 ± 1.1^b^	4.6 ± 3.0^c^	<0.01
Relative [% daily monounsaturated fats intake]	100	23.7 ± 19.0^a^	22.1 ± 16.9^a^	8.5 ± 10.6^b^	48.6 ± 24.4^c^	<0.01
**Polyunsaturated fats intake**						
Absolute [g]	10.0 ± 5.5	1.2 ± 1.2^a^	4.8 ± 4.1^b^	1.0 ± 1.0^c^	3.4 ± 3.2^d^	<0.01
Absolute [% daily energy intake]	7.1 ± 3.0	0.9 ± 0.9^a^	3.4 ± 2.7^b^	0.7 ± 0.6^c^	2.4 ± 2.0^d^	<0.01
Relative [% daily polyunsaturated fats intake]	100	14.0 ± 13.0^a^	42.9 ± 24.7^b^	12.2 ± 10.6^c^	35.2 ± 24.0^d^	<0.01
**Total sodium intake**						
Absolute [mg]	1,484.0 ± 670.4	191.5 ± 207.8^a^	489.7 ± 378.0^b^	223.6 ± 221.6 ^a^	579.3 ± 454.1^c^	<0.01
Absolute [mg/1,000 kcal intake]	1,216.6 ± 474.5	156.3 ± 156.0^a^	406.6 ± 317.2^b^	184.1 ± 177.3^a^	469.5 ± 386.2^c^	<0.01
Relative [% total sodium intake]	100	13.2 ± 12.1^a^	32.5 ± 18.3^b^	15.6 ± 14.6^c^	38.7 ± 20.2^d^	<0.01

*Quintiles were classified according to NOVA food groups among the Food Environment Chilean Cohort*.

*Values represent the mean ± standard deviation*.

* *One-way ANOVA across NOVA food groups; different letters indicate significant differences between groups. P-values <0.05 were considered statistically significant*.

**Figure 1:**
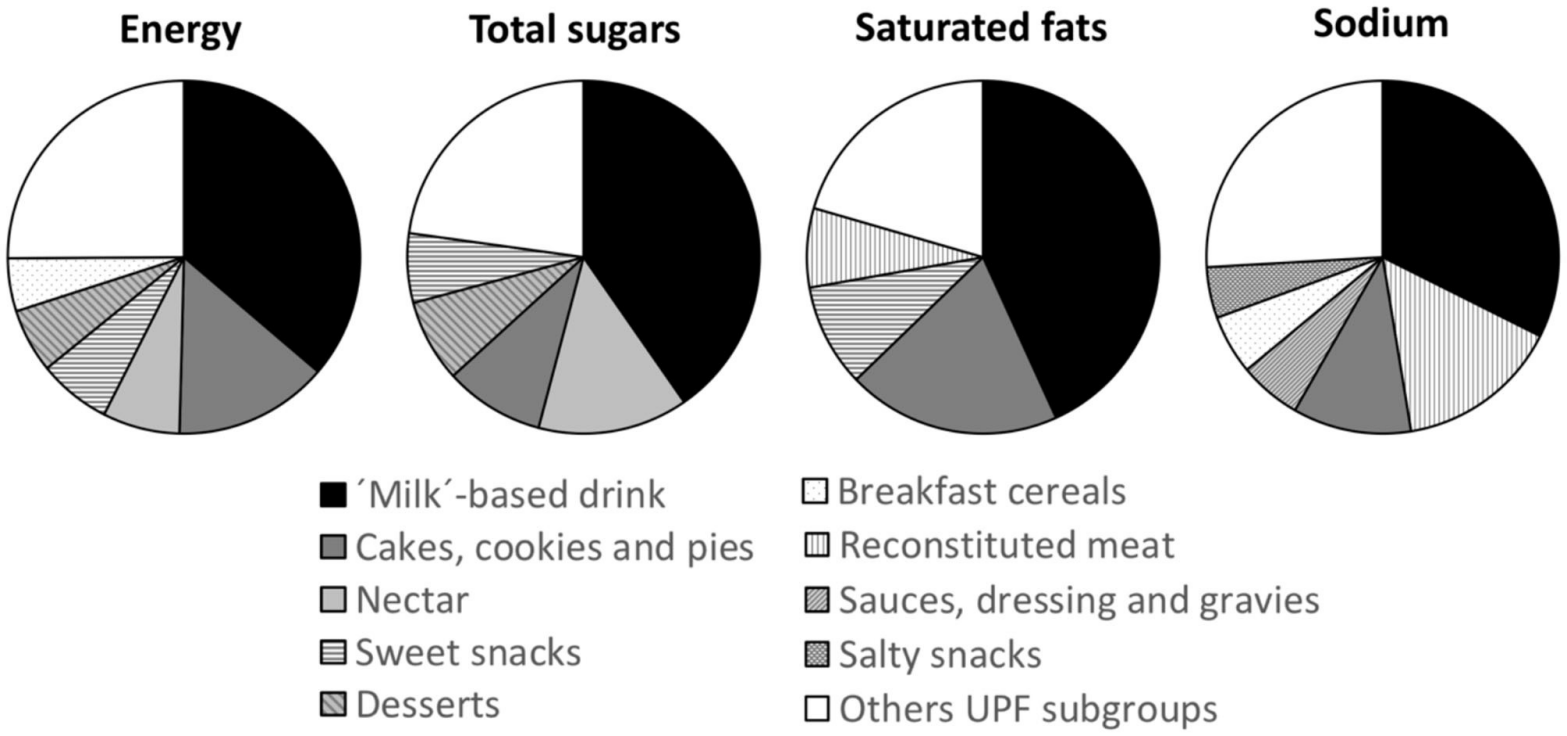
Main food groups contributing to the intake of energy, total sugars, saturated fats, and sodium among ultra-processed food (UPF). Only UPF subgroups contributing to 75% of the intake of energy, total sugars, saturated fats, and sodium are specified.

The details of the dietary share of energy, total sugars, saturated fats, and sodium of the different food subgroups within the UPF group are available in Figure 1 and Supplementary Tables 1–4. Within UPF, milk-based drinks were the primary source of energy (18%), total sugars (30%), saturated fats (24%), and sodium (13%). Cakes, cookies, and pies also contributed importantly to the UPF consumption of NCD-related nutrients: energy (7%), total sugars (7%), saturated fats (11%), and sodium (4%); as well as sweet snacks (energy (3%), total sugars (5%), saturated fats (5%), except in the case of sodium). Primary food sources of energy and total sugars also included nectar (4 and 10%, respectively) and desserts (3 and 6%, respectively), while sodium reconstituted meats (6%), sauces, dressing, and gravies (2%), and breakfast cereals (2%) also played a role. The details of the dietary shares of energy, total sugars, saturated fats, and sodium among subgroups of NOVA groups 1–3 are also displayed in Supplementary Tables 1–4.

Preschoolers were classified into five groups according to UPF intake: the mean dietary share of UPF increased in every quintile, at 24% in quintile 1, 40% in quintile 2, 50% in quintile 3, 60% in quintile 4, and 74% in quintile 5. Table 3 shows the sociodemographic characteristics, weight status, and energy intake according to NOVA food groups across the quintiles of UPF consumption. No difference in sociodemographic or anthropometric characteristic was observed by UPF quintile (*p* > 0.05); however, as expected, the dietary shares of every NOVA group differed significantly. Children in the first quintile consumed an average of 61% of their energy from minimally processed foods and culinary ingredients, whereas, only 21% of energy intake among children in the fifth quintile came from groups 1 and 2.

**Table 3:** Sociodemographic characteristics, weight status, and dietary share of NOVA food groups, by quintiles of UPF in the diet of the Food Environment Chilean Cohort (*n* = 960).

	**Q1: 1st quintile**	**Q2: 2nd quintile**	**Q3: 3rd quintile**	**Q4: 4th quintile**	**Q5: 5th quintile**	***p*-value**
	***n* = 192**	***n* = 192**	***n* = 192**	***n* = 192**	***n* = 192**	
Female, *n* (%)	101 (52.6)	101 (52.6)	96 (50.0)	99 (51.6)	101 (52.6)	0.98^†^
Age [years], mean ± SD	4.8 ± 0.5	4.7 ± 0.5	4.7 ± 0.6	4.8 ± 0.5	4.7 ± 0.5	0.66^¥^
BMI [z score], mean ± SD^*^	1.0 ± 1.3	1.0 ± 1.3	1.1 ± 1.1	1.0 ± 1.2	1.0 ± 1.1	0.99^¥^
Weight status, *n* (%)^*^						
Normal weight (< −1 to 1 SD)	100 (52.1)	108 (56.3)	99 (51.6)	101 (52.6)	100 (52.4)	
Overweight (1 to 1.9 SD)	56 (29.2)	50 (26.0)	59 (30.7)	55 (28.7)	56 (29.3)	0.99^†^
Obese (≥2 SD)	36 (18.8)	34 (17.7)	34 (17.7)	36 (18.8)	35 (18.3)	
Mother's education level, *n* (%)						
Low (<8 years of schooling)	16 (8.3)	15 (7.8)	12 (6.3)	14 (7.3)	13 (6.8)	
Medium (8–12 years of schooling)	96 (50.0)	107 (55.7)	98 (51.0)	106 (55.2)	92 (47.9)	0.76^†^
High (>12 years of schooling)	80 (41.7)	70 (36.5)	82 (42.7)	72 (37.5)	87 (45.3)	
Total energy intake according to NOVA food groups, mean ± SD						
Unprocessed or minimally processed foods [% of energy]	49.4 ± 14.3^a^	38.0 ± 10.1^b^	32.3 ± 9.2^c^	25.6 ± 8.0^d^	16.9 ± 7.5^e^	<0.01^¥^
Processed culinary ingredients [% of energy]	11.9 ± 6.9^a^	9.7 ± 6.3^b^	7.8 ± 4.6^c^	6.4 ± 4.2^c^	3.9 ± 3.1^d^	<0.01^¥^
Processed foods [% of energy]	15.1 ± 12.9^a^	12.9 ± 9.9^a,b^	10.4 ± 8.6^b,c^	8.4 ± 8.0^c^	5.3 ± 6.1^d^	<0.01^¥^
Ultra-processed foods [% of energy]	23.6 ± 8.1^a^	39.5 ± 3.0^b^	49.5 ± 2.8^c^	59.5 ± 3.2^d^	73.9 ± 7.8^e^	<0.01^¥^

*UPF, Ultra-processed foods*.

* *N = 191 in the fifth quintile with anthropometric data*.

† *Chi-squared test across UPF intake quintiles (i.e., 1st quintile, 2nd quintile, 3rd quintile, 4th quintile, and 5th quintile); different letters indicate significant differences between quintiles*.

¥ *One-way ANOVA across intake UPF quintiles (i.e., 1st quintile, 2nd quintile, 3rd quintile, 4th quintile, and 5th quintile); different letters indicate significant differences between quintiles. P-values <0.05 were considered statistically different*.

The adjusted intakes of energy and different nutrients by quintiles of UPF intake are presented in Table 4. Compared to the quintile with the lowest UPF intake, participants from other quintiles had significantly greater intakes of energy, saturated fats, monounsaturated fats, total carbohydrates, total sugars, and vitamin D. However, participants with a greater UPF dietary share had significantly lower intakes of proteins, polyunsaturated fats, fiber, sodium, zinc, vitamin A, and folate than the reference group (i.e., the first quintile). The intakes of total fats, iron, calcium, vitamin C, and vitamin B_12_ were not associated with UPF intake in this sample.

**Table 4:** Intake of energy, nutrients of concern, and nutrients for promotion by dietary contribution of UPF in the diet of the Food Environment Chilean Cohort (*n* = 959).

	**Q1: 1st quintile**	**Q2: 2nd quintile**	**Q3: 3rd quintile**	**Q4: 4th quintile**	**Q5: 5th quintile**
	***n* = 192**	***n* = 192**	***n* = 192**	***n* = 192**	***n* = 191**
Energy [kcal]	1,088.9 ± 29.9	1,214.1 ± 29.9^*^	1,249.9 ± 29.9^*^	1,248.2 ± 29.9^*^	1,253.9 ± 30.0^*^
Protein [% of energy]	15.9 ± 0.2	14.3 ± 0.2^*^	13.9 ± 0.2^*^	12.9 ± 0.2^*^	12.1 ± 0.2^*^
Total fats [% of energy]	28.8 ± 0.6	28.4 ± 0.6	28.9 ± 0.6	28.4 ± 0.6	30.2 ± 0.6
Saturated fats [% of energy]	9.1 ± 0.3	9.0 ± 0.3	9.3 ± 0.3	9.5 ± 0.3	10.7 ± 0.3^*^
Trans fats [% of energy]	0.2 ± 0.0	0.2 ± 0.0	0.2 ± 0.0	0.2 ± 0.0	0.2 ± 0.0
Monounsaturated fats [% of energy]	8.8 ± 0.2	8.7 ± 0.2	9.1 ± 0.2	9.2 ± 0.2	9.6 ± 0.2^*^
Polyunsaturated fats [% of energy]	7.4 ± 0.2	7.0 ± 0.2	7.1 ± 0.2	6.7 ± 0.2^*^	6.4 ± 0.2^*^
Carbohydrates [% of energy]	55.4 ± 0.6	58.7 ± 0.6^*^	57.4 ± 0.6^*^	59.5 ± 0.6^*^	57.8 ± 0.6^*^
Total sugars [% of energy]	22.1 ± 0.6	27.3 ± 0.6^*^	28.6 ± 0.6^*^	30.4 ± 0.6^*^	33.0 ± 0.6^*^
Fiber (g/1,000 kcal intake)	9.3 ± 0.3	9.0 ± 0.3	8.2 ± 0.3^*^	7.9 ± 0.3^*^	7.5 ± 0.3^*^
Sodium [mg/1,000 kcal intake]	1,299.3 ± 33.1	1,145.7 ± 33.2^*^	1,178.2 ± 33.2^*^	1,083.9 ± 33.1^*^	1,030.7 ± 33.3^*^
Iron [mg/1,000 kcal intake]	6.7 ± 0.2	7.0 ± 0.2	7.2 ± 0.2	7.2 ± 0.2	7.2 ± 0.2
Calcium [mg/1,000 kcal intake]	621.0 ± 20.3	595.9 ± 20.3	645.0 ± 20.3	630.5 ± 20.3	628.8 ± 20.4
Zinc [mg/1,000 kcal intake]	5.4 ± 0.2	5.2 ± 0.2	5.2 ± 0.2	5.0 ± 0.2^*^	5.0 ± 0.2^*^
Vitamin A [IU/1,000 kcal intake]	3,321.1 ± 208.5	3,571.3 ± 208.7	3,252.2 ± 208.6	2,851.6 ± 208.5	2,074.6 ± 209.5^*^
Vitamin D [IU/1,000 kcal intake]	40.2 ± 7.7	56.2 ± 7.7	80.4 ± 7.7^*^	76.6 ± 7.7^*^	90.3 ± 7.7^*^
Vitamin C [mg/1,000 kcal intake]	40.8 ± 2.8	45.3 ± 2.8	41.2 ± 2.8	42.2 ± 2.8	42.1 ± 2.8
Folate [μg/1,000 kcal intake]	200.6 ± 7.8	184.2 ± 7.8	168.4 ± 7.8^*^	143.6 ± 7.8^*^	141.7 ± 7.8^*^
Vitamin B_12_ [μg/1,000 kcal intake]	2.3 ± 0.1	2.2 ± 0.1	2.3 ± 0.1	2.3 ± 0.1	2.2 ± 0.1

*UPF, Ultra-processed food*.

*Values represent the adjusted mean ± standard error from predictive margin multivariate regression models*.

*Multivariate regression models considered quintiles of UPF intake as independent variables (reference group: 1st quintile) and sex, age, and weight status of preschoolers (three categories), mother's education (three categories), and day of the dietary recall (i.e., weekday vs. weekend) as covariates*.

* *P-values <0.05 from multivariate regression models, compared to 1st quintile of UPF intake (reference group)*.

## Discussion

The results of this study showed that UPF accounted for an important proportion of energy (~50%), total sugars (76%), saturated fats (55%), and sodium (~40%) intake among preschoolers from a longitudinal study in Santiago, Chile. UPF consumption was independently associated with the nutrient composition of the diet (i.e., greater intake of energy and nutrient of concern such as carbohydrates, total sugars, saturated and trans fats, and lower intake of nutrients for promotion, such as proteins, zinc, vitamin A, folate, and fiber.

Previous reports on the consumption of UPF in children are limited but in general, have shown a high dietary share of these products. In Belgium, the reported intake was 33% of daily energy for children and 29% for adolescents (2014–2015) (15); in Chile it was 38% for children aged 2–19 years (dietary data from 2010) (20), in Australia 53% (dietary data from 2011–2012) (9), 55% in Canada (dietary data from 2004) (6), and 65% in the United States of America (USA) (2009–2014) (14). Data from two Brazilian preschooler samples (the Pelotas cohort) showed a 36% energy intake from UPF among children between 2 and 6 years of age (dietary data from 2008) (37), reaching 40% among 6-year-old children (dietary data from 2004) (38). The age of participants, year of dietary collection, and the type of culinary culture may explain the differences in the dietary shares of UPF but overall, the data reflect the relevance that these type of foods have on the diet of children from countries facing advance stages of the nutrition transition.

Other studies in the general population have also reported an association between UPF intake and an unhealthier nutrient composition of diet. According to the 2010 Chilean National Dietary Survey, UPF consumption was positively associated with the intake of nutrients of concern, specifically energy density, free sugars, total fats, saturated fats, and trans fats, and negatively associated with the intake of potassium and fiber (8). Data derived from the 2004 Canadian dietary survey showed that UPF intake was associated with diets with greater energy density, carbohydrates, free sugars, total and saturated fats but lower contents of proteins, fiber, vitamins A, C, D, B6, and B_12_, niacin, thiamine, and riboflavin as well as zinc, iron, magnesium, calcium, phosphorus, and potassium (6). According to nationally representative surveys, children from the USA, Australia, and Chile reporting higher UPF intake showed greater intakes of free or added sugars (9, 14, 20), while those from Belgium not meeting the dietary goals for fruits and vegetables, sodium, saturated, and trans fats had higher UPF intake (15). Other studies in specific pediatric populations have reported the impact of UPF intake on the nutrient composition of their diet. Among adolescents from the Pelotas cohort, a greater UPF intake was associated with lower inadequacy of selenium and vitamin B1 (39), whereas the combined intake of processed and UPF was associated with lower intakes of vitamins A and E, zinc, proteins, polyunsaturated and trans fats, and fiber along with greater intake of total and saturated fats, cholesterol, sugars, calcium, and sodium in children aged 5–12 years from Colombia (40).

While most reports are consistent regarding the impact of UPF consumption on a greater intake of nutrients of concern (except for sodium) and lower consumption of fiber, the available information regarding micronutrient intake remains controversial. The latter could be explained by country-to-country differences in micronutrient fortification policies and the quality (e.g., dietary diversity) of diets based on food groups with a lower degree of processing (i.e., NOVA groups 1–3). We did observe a positive association between UPF and vitamin D, likely due to the fact that several of the main UPF food groups are fortified with vitamin D in the USA. Several authors have highlighted the risks associated with promoting the intake of vitamins in UPF foods and whether this should be a matter of concern and have more active monitoring (41). In the case of sodium intake, the discrepancies can be explained by the difficulty of assessing sodium intake (42) and by differences between countries on the other relevant dietary sources of sodium. For instance, in the current study, UPF was the main NOVA group contributor to sodium intake (39% daily intake); however, table salt and fresh bread accounted for 45% of daily sodium intake.

Given that the first years of life, including the preschool period, are relevant in establishing future food preferences (43, 44), high UPF consumption could translate into a notable future increase in UPF intake in the adult population. In turn, greater UPF intake could lead to overeating and weight gain (45) as well as the development of NCDs (10–12) and greater mortality (13). One method to shift this trend is to modify aspects of the food environment that facilitate the consumption of these products, such as availability, marketing, and price. The recent implementation of the Food Labeling and Marketing Law in Chile (24), in addition to other initiatives (25), may mark an important shift in these tendencies (46, 47). Newly implemented measures include increasing the price of sugar-sweetened beverages, the required use of warning labels when the content of nutrients of concern exceeds established values, the prohibition of selling or freely offering foods that exceed established cutoffs in schools, and restrictions on marketing directed toward children. While these measures are not specifically directed at UPF intake, there is an important overlap between these products and those subject to the new regulations (i.e., foods that, as part of processing, have added sodium, sugars, or saturated fats, which exceed established values) (24). This study serves as the basis for evaluating changes in UPF consumption by preschool-aged children and for assessing the nutrients of concern derived from UPF.

The present study has several limitations that are important to mention. The sample was limited to children from nurseries in low-middle-income neighborhoods in Southeast Santiago and may not represent preschool children from other socioeconomic backgrounds in Santiago or across Chile. Only one 24 h survey was used for the analysis, which does not represent usual dietary intake. Furthermore, given the lack of a Chilean database for the chemical composition of foods and beverages, the USDA database for chemical compositions was used. The foods recorded in the USDA database may present important differences in the contents of some nutrients as compared to foods available in Chile, particularly for packaged products (i.e., with some degree of processing). To minimize this limitation, foods listed by the USDA were selected according to the similarity of nutritional information (i.e., energy/calories, macronutrients, and nutrients of concern) provided by the Chilean table of food compositions and, in the case of packaged products, by the nutritional contents reported by manufacturers. As the selection of foods did not consider other nutrients or factors, the results regarding micronutrients and fiber should be interpreted based on this important limitation. This may be especially relevant for vitamin D, which is fortified among dairy products in the USA but not in Chile. Finally, we did not have any biomarker to more accurately assess the diet, which could be especially important for sodium intake. A strength of this study was that all information was collected by a team of trained professionals using standardized methods and supplemented by an atlas to estimate portions, with photographs of foods, and with locally relevant recipes, which supports the precision of the reported results; moreover, we also had access to the recipes used by the school feeding program.

In conclusion, in 4–6-year-old preschoolers from Santiago, Chile, UPF were an important source of energy and nutrients of concern such as total sugars, saturated fats, and sodium. Children with a greater dietary share of UPF had diets characterized by greater content of energy, saturated fats, carbohydrates, and total sugars, and a lower content of fiber, zinc, vitamin A, and folate. Early dietary behaviors might influence long-term dietary behaviors and health status and therefore, improving children's diet should be prioritized on the nutrition agenda. Our results highlight the need of implementing actions to decrease UPF consumption while promoting the consumption of minimally processed and traditionally prepared foods; specific actions targeting sodium might also be required for achieving healthier diets.

## Data Availability Statement

The datasets presented in this article are not readily available because of funder's restrictions. Requests to access the datasets should be directed to mreyes@inta.uchile.cl.

## Ethics Statement

The studies involving human participants were reviewed and approved by Ethics Committee of the Institute of Nutrition and Food Technology (INTA), University of Chile, and the Institutional Review Board of the University of North Carolina at Chapel Hill. Written informed consent to participate in this study was provided by the participants' legal guardian/next of kin.

## Author Contributions

MR, CC, and LT contributed to the overall study conception and design, contributed to funding acquisition, study execution, and coordination. CA and GC classified the food and beverages according to the NOVA groups and subgroups. CA analyzed and interpreted the data with assistance from MR and CC and wrote the original draft of the manuscript, which the other co-authors reviewed and edited. MR has full access to all the data in the study and has final responsibility for the decision to submit for publication. All authors approved the final version of the manuscript before submission.

## Conflict of Interest

The authors declare that the research was conducted in the absence of any commercial or financial relationships that could be construed as a potential conflict of interest.
