# Supplementary material for: Ultra-Processed Food Consumption Among Chilean Preschoolers Is Associated With Diets Promoting Non-communicable Diseases
Source: Front Nutr. 2021 Mar 26;8:601526. doi: 10.3389/fnut.2021.601526 (PMC8032866; doi:10.3389/fnut.2021.601526)
Supplement: Supplementary file 1 [file Table_1.DOCX]

| **Table 1. Distribution of total energy intake according to NOVA food group and subgroups in the diet FeChic Cohort (n=960)** | | | | | | |
| --- | --- | --- | --- | --- | --- | --- |
|  | Mean energy intake | | | | | |
|  | Absolute (Kcal) | | | Relative (% of daily energy intake) | | |
| NOVA food group | Mean | SD | SE | Mean | SD | SE |
| **Total unprocessed or minimally processed foods** | **397** | **209** | **6.7** | **32.4** | **14.9** | **0.5** |
| Others unprocessed or minimally processed food^1^ | 79 | 120 | 3.9 | 6.1 | 9 | 0.3 |
| Cereals | 59 | 81 | 2.6 | 5 | 6.6 | 0.2 |
| Milk and plain yoghurt | 56 | 104 | 3.3 | 4.7 | 8.5 | 0.3 |
| Meat | 51 | 62 | 2 | 4.2 | 5.1 | 0.2 |
| Fruits | 35 | 47 | 1.5 | 2.9 | 4 | 0.1 |
| Roots and tubers | 33 | 54 | 1.7 | 2.8 | 4.5 | 0.1 |
| Vegetables | 27 | 37 | 1.2 | 2.2 | 2.8 | 0.1 |
| Legumes | 26 | 71 | 2.3 | 2 | 5.4 | 0.2 |
| Eggs | 24 | 47 | 1.5 | 2 | 3.9 | 0.1 |
| Natural fruit juices | 4 | 21 | 0.7 | 0.3 | 1.7 | 0.1 |
| Fish and seafood | 2 | 14 | 0.5 | 0.2 | 1.2 | 0 |
| Dried fruits | 2 | 16 | 0.5 | 0.1 | 1.2 | 0 |
| Plain water | 0 | 0 | 0 | 0 | 0 | 0 |
| **Total processed culinary ingredients** | **99** | **80** | **2.6** | **7.9** | **5.9** | **0.2** |
| Plant oils | 76 | 66 | 2.1 | 6 | 4.9 | 0.2 |
| Table sugar | 12 | 30 | 1 | 1 | 2.4 | 0.1 |
| Animal fats | 11 | 22 | 0.7 | 0.9 | 1.7 | 0.1 |
| Table salt | 0 | 0 | 0 | 0 | 0 | 0 |
| Others processed culinary ingredients^2^ | 0 | 3 | 0.1 | 0 | 0.3 | 0 |
| **Total processed foods** | **127** | **125** | **4.0** | **10.4** | **9.9** | **0.3** |
| Breads (fresh unpackaged) | 99 | 104 | 3.3 | 8.3 | 8.6 | 0.3 |
| Cheese | 16 | 40 | 1.3 | 1.2 | 3.1 | 0.1 |
| Ham and other salted, smoked or canned meat or fish | 5 | 22 | 0.7 | 0.4 | 1.8 | 0.1 |
| Others processed foods^3^ | 5 | 34 | 1.1 | 0.4 | 2.4 | 0.1 |
| Vegetables, fruits and other plant foods preserved in brine or syrup | 2 | 12 | 0.4 | 0.1 | 0.9 | 0 |
| **Total ultra-processed foods** | **618** | **318** | **10.3** | **49.2** | **18.0** | **0.6** |
| ´Milk´-based drink | 215.6 | 163.4 | 5.3 | 17.9 | 12.9 | 0.4 |
| Cakes, cookies and pies | 93.9 | 157.5 | 5.1 | 7.0 | 11.0 | 0.4 |
| Nectar | 43.6 | 74.4 | 2.4 | 3.5 | 5.8 | 0.2 |
| Sweet snacks | 42.9 | 103.6 | 3.3 | 3.3 | 7.0 | 0.2 |
| Desserts | 33.7 | 72.0 | 2.3 | 2.9 | 6.4 | 0.2 |
| Breakfast cereals | 30.1 | 55.5 | 1.8 | 2.4 | 4.4 | 0.1 |
| Reconstituted meat | 26.0 | 63.0 | 2.0 | 2.0 | 4.8 | 0.2 |
| Salty snacks | 22.9 | 78.9 | 2.5 | 1.7 | 5.3 | 0.2 |
| Sauces, dressing and gravies | 18.6 | 33.6 | 1.1 | 1.5 | 2.7 | 0.1 |
| Soft drinks, carbonated | 17.8 | 44.2 | 1.4 | 1.4 | 3.3 | 0.1 |
| Others ultra-processed foods^4^ | 17.1 | 41.0 | 1.3 | 1.4 | 3.1 | 0.1 |
| Breads (packaged) | 15.5 | 53.2 | 1.7 | 1.1 | 3.8 | 0.1 |
| Nectar reduced calories | 6.7 | 25.5 | 0.8 | 0.6 | 2.2 | 0.1 |
| Sandwiches & hamburgers on bun (ready to eat/heat) | 8.0 | 47.1 | 1.5 | 0.5 | 3.1 | 0.1 |
| Refreshers instant juice | 6.3 | 22.9 | 0.7 | 0.5 | 1.8 | 0.1 |
| Ice cream and ice pops | 5.2 | 28.7 | 0.9 | 0.4 | 2.3 | 0.1 |
| Pizza (ready to eat/heat) | 4.8 | 33.4 | 1.1 | 0.4 | 2.6 | 0.1 |
| Frozen and shelf-stable plate meals | 4.5 | 31.7 | 1.0 | 0.4 | 2.4 | 0.1 |
| Instant and canned soups | 2.4 | 19.6 | 0.6 | 0.2 | 2.1 | 0.1 |
| Refreshers instant juice reduced calories | 1.9 | 5.5 | 0.2 | 0.2 | 0.5 | 0.0 |
| ´Fruit´ drinks/sweetened ´water´ | 1.0 | 8.5 | 0.3 | 0.1 | 0.8 | 0.0 |
| **Total** | **1240** | **392** | **12.7** | **100** | | |

^1^Others unprocessed or minimally processed foods: spices, coffee, tea, herbs and pasta

^2^Others processed culinary ingredients: honeybee and vinegar

^3^Others processed foods: flavored baby food, french fries and salted or sugared dried fruits

^4^Other ultra-processed foods: artificial sweeteners, artificial flavoring and soy-based drinks

SD: standard deviation

SE: standard error

| **Table 2. Distribution of total sugars intake according to NOVA food group in the diet FeChic Cohort (n=960)** | | | | | | |
| --- | --- | --- | --- | --- | --- | --- |
|  | Mean total sugars intake | | | | | |
|  | Absolute (g) | | | Relative (% of daily total sugars intake) | | |
| NOVA food group | Mean | SD | SE | Mean | SD | SE |
| **Total unprocessed or minimally processed foods** | **15** | **14** | **0.5** | **18.6** | **17.6** | **0.6** |
| Fruits | 6 | 8.3 | 0.3 | 7.4 | 10.5 | 0.3 |
| Milk and plain yoghurt | 5.1 | 9.1 | 0.3 | 6.3 | 11.6 | 0.4 |
| Vegetables | 1.8 | 1.8 | 0.1 | 2.3 | 2.7 | 0.1 |
| Natural fruit juices | 0.7 | 4.3 | 0.1 | 0.8 | 4.5 | 0.1 |
| Roots and tubers | 0.4 | 0.6 | 0 | 0.5 | 1 | 0 |
| Legumes | 0.3 | 1 | 0 | 0.4 | 1.3 | 0 |
| Others unprocessed or minimally processed food^1^ | 0.3 | 0.9 | 0 | 0.4 | 1.4 | 0 |
| Cereals | 0.2 | 0.6 | 0 | 0.3 | 1.1 | 0 |
| Eggs | 0.1 | 0.3 | 0 | 0.2 | 0.4 | 0 |
| Plain water | 0 | 0 | 0 | 0 | 0 | 0 |
| Dried fruits | 0 | 0.1 | 0 | 0 | 0.2 | 0 |
| Meat | 0 | 0 | 0 | 0 | 0.1 | 0 |
| Fish and seafood | 0 | 0 | 0 | 0 | 0 | 0 |
| **Total processed culinary ingredients** | **3.2** | **7.6** | **0.2** | **3.7** | **8.4** | **0.3** |
| Table sugar | 3.1 | 7.6 | 0.2 | 3.6 | 8.4 | 0.3 |
| Others processed culinary ingredients^2^ | 0.1 | 0.8 | 0 | 0.1 | 1.1 | 0 |
| Plant oils | 0 | 0.1 | 0 | 0 | 0.2 | 0 |
| Animal fats | 0 | 0.2 | 0 | 0 | 0 | 0 |
| Table salt | 0 | 0 | 0 | 0 | 0 | 0 |
| **Total processed foods** | **1.6** | **3.1** | **0.1** | **2.2** | **4.5** | **0.1** |
| Breads (fresh unpackaged) | 1.1 | 1.2 | 0 | 1.5 | 2.5 | 0.1 |
| Vegetables, fruits and other plant foods preserved in brine or syrup | 0.4 | 2.7 | 0.1 | 0.4 | 2.7 | 0.1 |
| Others processed foods^3^ | 0.1 | 1 | 0 | 0.2 | 2 | 0.1 |
| Cheese | 0.1 | 0.2 | 0 | 0.1 | 0.3 | 0 |
| Ham and other salted, smoked or canned meat or fish | 0 | 0 | 0 | 0 | 0 | 0 |
| **Total ultra-processed foods** | **68.3** | **35.5** | **1.1** | **75.5** | **19.9** | **0.6** |
| ´Milk´-based drink | 25.8 | 19.7 | 0.6 | 30.4 | 21.6 | 0.7 |
| Nectar | 10.5 | 18.0 | 0.6 | 10.4 | 16.6 | 0.5 |
| Cakes, cookies and pies | 6.6 | 11.7 | 0.4 | 6.9 | 11.3 | 0.4 |
| Desserts | 5.4 | 12.4 | 0.4 | 5.7 | 12.7 | 0.4 |
| Sweet snacks | 4.6 | 11.1 | 0.4 | 4.8 | 10.4 | 0.3 |
| Soft drinks, carbonated | 4.3 | 10.7 | 0.3 | 4.5 | 10.5 | 0.3 |
| Others ultra-processed foods^4^ | 3.4 | 7.6 | 0.2 | 3.5 | 6.9 | 0.2 |
| Breakfast cereals | 1.9 | 3.8 | 0.1 | 2.3 | 4.7 | 0.2 |
| Refreshers instant juice | 1.6 | 5.8 | 0.2 | 1.8 | 6.4 | 0.2 |
| Nectar reduced calories | 1.4 | 5.4 | 0.2 | 1.7 | 6.5 | 0.2 |
| Sauces, dressing and gravies | 1.2 | 3.4 | 0.1 | 1.4 | 4.0 | 0.1 |
| Ice cream and ice pops | 0.6 | 2.7 | 0.1 | 0.7 | 3.3 | 0.1 |
| Breads (packaged) | 0.3 | 1.5 | 0.0 | 0.3 | 1.5 | 0.0 |
| Reconstituted meat | 0.2 | 0.7 | 0.0 | 0.3 | 0.9 | 0.0 |
| ´Fruit´ drinks/sweetened ´water´ | 0.2 | 2.1 | 0.1 | 0.3 | 2.3 | 0.1 |
| Instant and canned soups | 0.1 | 0.4 | 0.0 | 0.2 | 3.3 | 0.1 |
| Salty snacks | 0.1 | 0.3 | 0.0 | 0.1 | 0.4 | 0.0 |
| Pizza (ready to eat/heat) | 0.1 | 0.4 | 0.0 | 0.1 | 0.6 | 0.0 |
| Frozen and shelf-stable plate meals | 0.0 | 0.0 | 0.0 | 0.0 | 0.0 | 0.0 |
| Sandwiches & hamburgers on bun (ready to eat/heat) | 0.0 | 0.2 | 0.0 | 0.0 | 0.3 | 0.0 |
| Refreshers instant juice reduced calories | 0.0 | 0.2 | 0.0 | 0.0 | 0.3 | 0.0 |
| **Total** | **88.2** | **35.9** | **1.2** | **100** | | |
| ^1^Others unprocessed or minimally processed foods: spices, coffee, tea, herbs and pasta | | | | | | |
| ^2^Others processed culinary ingredients: honeybee and vinegar | | | | | | |
| ^3^Others processed foods: flavored baby food, french fries and salted or sugared dried fruits  4 Other ultra-processed foods: artificial sweeteners, artificial flavoring and soy-based drink | | | | | | |

SD: standard deviation

SE: standard error

| **Table 3. Distribution of total saturated fats intake according to NOVA food group in the diet FeChic Cohort (n=960)** | | | | | | |
| --- | --- | --- | --- | --- | --- | --- |
|  | Mean saturated fats intake | | | | | |
|  | Absolute (g) | | | Relative (% of daily saturated fats intake) | | |
| NOVA food group | Mean | SD | SE | Mean | SD | SE |
| **Total unprocessed or minimally processed foods** | **3.2** | **3.6** | **0.1** | **22.9** | **20.4** | **0.7** |
| Milk and plain yoghurt | 1.6 | 3.3 | 0.1 | 9.6 | 18 | 0.6 |
| Meat | 0.7 | 1.2 | 0 | 6.2 | 9.5 | 0.3 |
| Eggs | 0.5 | 1 | 0 | 4.3 | 9 | 0.3 |
| Vegetables | 0.1 | 0.3 | 0 | 1 | 2.9 | 0.1 |
| Others unprocessed or minimally processed food^1^ | 0.1 | 0.1 | 0 | 0.7 | 1.3 | 0 |
| Cereals | 0 | 0.1 | 0 | 0.4 | 0.8 | 0 |
| Dried fruits | 0 | 0.2 | 0 | 0.2 | 1.5 | 0.1 |
| Fruits | 0 | 0 | 0 | 0.2 | 0.4 | 0 |
| Legumes | 0 | 0.1 | 0 | 0.2 | 0.6 | 0 |
| Roots and tubers | 0 | 0 | 0 | 0.1 | 0.2 | 0 |
| Fish and seafood | 0 | 0.1 | 0 | 0.1 | 0.8 | 0 |
| Plain water | 0 | 0 | 0 | 0 | 0 | 0 |
| Natural fruit juices | 0 | 0 | 0 | 0 | 0.1 | 0 |
| **Total processed culinary ingredients** | **1.7** | **1.8** | **0.1** | **13.7** | **13.2** | **0.4** |
| Plant oils | 1.1 | 0.9 | 0 | 8.8 | 8.5 | 0.3 |
| Animal fats | 0.7 | 1.4 | 0.1 | 4.9 | 9.8 | 0.3 |
| Table sugar | 0 | 0 | 0 | 0 | 0 | 0 |
| Table salt | 0 | 0 | 0 | 0 | 0 | 0 |
| Others processed culinary ingredients^2^ | 0 | 0 | 0 | 0 | 0 | 0 |
| **Total processed foods** | **1.2** | **2.1** | **0.1** | **8.2** | **12.9** | **0.4** |
| Cheese | 0.8 | 1.9 | 0.1 | 4.7 | 11.3 | 0.4 |
| Breads (fresh unpackaged) | 0.3 | 0.3 | 0 | 2.6 | 4.4 | 0.1 |
| Others processed foods^3^ | 0.1 | 0.5 | 0 | 0.6 | 3.9 | 0.1 |
| Ham and other salted, smoked or canned meat or fish | 0 | 0.2 | 0 | 0.4 | 1.8 | 0.1 |
| Vegetables, fruits and other plant foods preserved in brine or syrup | 0 | 0 | 0 | 0 | 0 | 0 |
| **Total ultra-processed foods** | **7.8** | **5.7** | **0.2** | **55.1** | **25.1** | **0.8** |
| ´Milk´-based drink | 3.1 | 2.9 | 0.1 | 23.8 | 19.8 | 0.6 |
| Cakes, cookies and pies | 1.7 | 3.2 | 0.1 | 10.8 | 17.3 | 0.6 |
| Sweet snacks | 0.9 | 2.7 | 0.1 | 5.2 | 12.5 | 0.4 |
| Reconstituted meat | 0.6 | 1.4 | 0.0 | 4.0 | 9.8 | 0.3 |
| Sauces, dressing and gravies | 0.4 | 0.8 | 0.0 | 3.0 | 6.5 | 0.2 |
| Desserts | 0.3 | 1.3 | 0.0 | 2.3 | 6.9 | 0.2 |
| Salty snacks | 0.2 | 0.7 | 0.0 | 1.5 | 5.5 | 0.2 |
| Sandwiches & hamburgers on bun (ready to eat/heat) | 0.2 | 1.0 | 0.0 | 1.0 | 5.6 | 0.2 |
| Ice cream and ice pops | 0.1 | 1.1 | 0.0 | 0.7 | 5.1 | 0.2 |
| Others ultra-processed foods^4^ | 0.1 | 0.2 | 0.0 | 0.6 | 2.1 | 0.1 |
| Pizza (ready to eat/heat) | 0.1 | 0.5 | 0.0 | 0.5 | 3.9 | 0.1 |
| Breakfast cereals | 0.1 | 0.2 | 0.0 | 0.5 | 1.7 | 0.1 |
| Breads (packaged) | 0.1 | 0.2 | 0.0 | 0.4 | 2.1 | 0.1 |
| Frozen and shelf-stable plate meals | 0.0 | 0.3 | 0.0 | 0.4 | 2.8 | 0.1 |
| Instant and canned soups | 0.0 | 0.3 | 0.0 | 0.3 | 3.8 | 0.1 |
| Nectar | 0.0 | 0.0 | 0.0 | 0.1 | 0.4 | 0.0 |
| Soft drinks, carbonated | 0.0 | 0.0 | 0.0 | 0.0 | 0.0 | 0.0 |
| Refreshers instant juice | 0.0 | 0.0 | 0.0 | 0.0 | 0.0 | 0.0 |
| Refreshers instant juice reduced calories | 0.0 | 0.0 | 0.0 | 0.0 | 0.0 | 0.0 |
| Nectar reduced calories | 0.0 | 0.0 | 0.0 | 0.0 | 0.1 | 0.0 |
| ´Fruit´ drinks/sweetened ´water´ | 0.0 | 0.0 | 0.0 | 0.0 | 0.0 | 0.0 |
| **Total** | **13.9** | **6.8** | **0.2** | **100** | | |
| ^1^Others unprocessed or minimally processed foods: spices, coffee, tea, herbs and pasta | | | | | | |
| ^2^Others processed culinary ingredients: honeybee and vinegar | | | | | | |
| ^3^Others processed foods: flavored baby food, french fries and salted or sugared dried fruits  ^4^Other ultra-processed foods: artificial sweeteners, artificial flavoring and soy-based drinks | | | | | | |

SD: standard deviation

SE: standard error

| **Table 4. Distribution of total sodium intake according to NOVA food group in the diet FeChic Cohort (n=960)** | | | | | | |
| --- | --- | --- | --- | --- | --- | --- |
|  | Mean sodium intake | | | | | |
|  | Absolute (mg) | | | Relative (% of daily sodium intake) | | |
| NOVA food group | Mean | SD | SE | Mean | SD | SE |
| **Total unprocessed or minimally processed foods** | **191.5** | **207.8** | **6.7** | **13.2** | **12.1** | **0.4** |
| Milk and plain yoghurt | 47.5 | 86.2 | 2.8 | 3.6 | 7 | 0.2 |
| Eggs | 28.6 | 54.7 | 1.8 | 2 | 3.9 | 0.1 |
| Meat | 20.7 | 33.9 | 1.1 | 1.5 | 2.6 | 0.1 |
| Vegetables | 23.3 | 75.6 | 2.4 | 1.4 | 3.5 | 0.1 |
| Others unprocessed or minimally processed food^1^ | 20.4 | 67.8 | 2.2 | 1.3 | 4.4 | 0.1 |
| Plain water | 15.6 | 14.9 | 0.5 | 1.2 | 1.3 | 0 |
| Cereals | 19.4 | 100.4 | 3.2 | 1.2 | 6 | 0.2 |
| Roots and tubers | 11.6 | 76.7 | 2.5 | 0.6 | 3.6 | 0.1 |
| Fruits | 1 | 2 | 0.1 | 0.1 | 0.2 | 0 |
| Legumes | 1.4 | 16.9 | 0.5 | 0.1 | 0.8 | 0 |
| Fish and seafood | 1.5 | 10 | 0.3 | 0.1 | 0.7 | 0 |
| Natural fruit juices | 0.3 | 2.5 | 0.1 | 0 | 0.2 | 0 |
| Dried fruits | 0 | 0.4 | 0 | 0 | 0 | 0 |
| **Total processed culinary ingredients** | **489.7** | **378** | **12.2** | **32.5** | **18.3** | **0.6** |
| Table salt | 480.9 | 375.3 | 0 | 32 | 18.3 | 0 |
| Animal fats | 7.1 | 16.1 | 0.5 | 0.5 | 1.2 | 0 |
| Others processed culinary ingredients^2^ | 1.5 | 27.3 | 0 | 0.1 | 1.2 | 0 |
| Table sugar | 0.1 | 0.3 | 0 | 0 | 0 | 0 |
| Plant oils | 0.1 | 1.2 | 0 | 0 | 0.1 | 0 |
| **Total processed foods** | **223.6** | **221.6** | **7.2** | **15.6** | **14.6** | **0.5** |
| Breads (fresh unpackaged) | 183.5 | 194 | 6.3 | 13 | 13.2 | 0.4 |
| Cheese | 23.5 | 66.2 | 2.1 | 1.5 | 4 | 0.1 |
| Ham and other salted, smoked or canned meat or fish | 12.5 | 55.8 | 1.8 | 0.8 | 3.5 | 0.1 |
| Others processed foods^3^ | 3.5 | 21.1 | 0.7 | 0.3 | 1.7 | 0.1 |
| Vegetables, fruits and other plant foods preserved in brine or syrup | 0.6 | 10.9 | 0 | 0 | 0.5 | 0 |
| **Total ultra-processed foods** | **579.3** | **454.1** | **14.7** | **38.7** | **20.2** | **0.7** |
| ´Milk´-based drink | 161.0 | 135.4 | 4.4 | 12.5 | 11.1 | 0.4 |
| Reconstituted meat | 113.8 | 287.2 | 9.3 | 5.8 | 12.4 | 0.4 |
| Cakes, cookies and pies | 55.5 | 98.3 | 3.2 | 4.2 | 7.6 | 0.2 |
| Sauces, dressing and gravies | 35.7 | 89.9 | 2.9 | 2.2 | 4.9 | 0.2 |
| Breakfast cereals | 30.6 | 62.7 | 2.0 | 2.1 | 4.3 | 0.1 |
| Salty snacks | 27.1 | 91.9 | 3.0 | 1.8 | 6.1 | 0.2 |
| Desserts | 20.6 | 51.1 | 1.6 | 1.7 | 4.2 | 0.1 |
| Breads (packaged) | 24.3 | 89.3 | 2.9 | 1.4 | 5.3 | 0.2 |
| Instant and canned soups | 25.2 | 158.5 | 5.1 | 1.2 | 6.8 | 0.2 |
| Sweet snacks | 16.6 | 52.9 | 1.7 | 1.2 | 3.5 | 0.1 |
| Refreshers instant juice reduced calories | 12.1 | 35.3 | 1.1 | 0.9 | 2.6 | 0.1 |
| Sandwiches & hamburgers on bun (ready to eat/heat) | 14.0 | 107.6 | 3.5 | 0.7 | 4.9 | 0.2 |
| Frozen and shelf-stable plate meals | 8.8 | 62.4 | 2.0 | 0.6 | 4.0 | 0.1 |
| Pizza (ready to eat/heat) | 9.1 | 62.2 | 2.0 | 0.6 | 3.7 | 0.1 |
| Nectar | 6.2 | 19.3 | 0.6 | 0.5 | 1.3 | 0.0 |
| Others ultra-processed foods^4^ | 8.0 | 66.4 | 2.1 | 0.5 | 2.8 | 0.1 |
| Soft drinks, carbonated | 3.2 | 9.0 | 0.3 | 0.2 | 0.6 | 0.0 |
| Ice cream and ice pops | 2.1 | 15.5 | 0.5 | 0.2 | 1.2 | 0.0 |
| Refreshers instant juice | 3.2 | 11.8 | 0.4 | 0.2 | 0.9 | 0.0 |
| Nectar reduced calories | 1.6 | 7.8 | 0.3 | 0.1 | 0.6 | 0.0 |
| ´Fruit´ drinks/sweetened ´water´ | 0.4 | 4.8 | 0.2 | 0.0 | 0.5 | 0.0 |
| **Total** | **1484** | **670.3** | **21.6** | **100** | | |
| ^1^Others unprocessed or minimally processed foods: spices, coffee, tea, herbs and pasta | | | | | | |
| ^2^Others processed culinary ingredients: honeybee and vinegar | | | | | | |
| ^3^Others processed foods: flavored baby food, french fries and salted or sugared dried fruits  ^4^Other ultra-processed foods: artificial sweeteners, artificial flavoring and soy-based drinks | | | | | | |

SD: standard deviation

SE: standard error
